# Molecular dynamics of seed priming at the crossroads between basic and applied research

**DOI:** 10.1007/s00299-023-02988-w

**Published:** 2023-02-13

**Authors:** Andrea Pagano, Anca Macovei, Alma Balestrazzi

**Affiliations:** 1 Department of Biology and Biotechnology ‘L. Spallanzani’, Via Ferrata 1, 27100 Pavia, Italy; 2 National Biodiversity Future Center (NBFC), 90133 Palermo, Italy

**Keywords:** Molecular hallmarks, Pre-germinative metabolism, Seed priming, Seed quality, Sustainability

## Abstract

**Key message:**

The potential of seed priming is still not fully exploited. Our limited knowledge of the molecular dynamics of seed pre-germinative metabolism is the main hindrance to more effective new-generation techniques.

**Abstract:**

Climate change and other recent global crises are disrupting food security. To cope with the current demand for increased food, feed, and biofuel production, while preserving sustainability, continuous technological innovation should be provided to the agri-food sector. Seed priming, a pre-sowing technique used to increase seed vigor, has become a valuable tool due to its potential to enhance germination and stress resilience under changing environments. Successful priming protocols result from the ability to properly act on the seed pre-germinative metabolism and stimulate events that are crucial for seed quality. However, the technique still requires constant optimization, and researchers are committed to addressing some key open questions to overcome such drawbacks. In this review, an update of the current scientific and technical knowledge related to seed priming is provided. The rehydration–dehydration cycle associated with priming treatments can be described in terms of metabolic pathways that are triggered, modulated, or turned off, depending on the seed physiological stage. Understanding the ways seed priming affects, either positively or negatively, such metabolic pathways and impacts gene expression and protein/metabolite accumulation/depletion represents an essential step toward the identification of novel seed quality hallmarks. The need to expand the basic knowledge on the molecular mechanisms ruling the seed response to priming is underlined along with the strong potential of applied research on primed seeds as a source of seed quality hallmarks. This route will hasten the implementation of seed priming techniques needed to support sustainable agriculture systems.

## Introduction

Plants and seeds are central in our life, being part of our cultures, religions, and medicines. The connection between seeds and humankind started with the domestication of wild species, when seeds were collected, sown, and then harvested from the best-performing plants, looking for the most favorable features. Since then, our knowledge of seeds has grown, revealing the impressive complexity of this biological system. Although this era provides researchers with breakthrough technological tools, many research questions are still open. Nowadays, seeds have gained immense economic value as the global seed market was worth 58.5 billion U.S. Dollars in 2020 and it is estimated to grow up to 105.3 billion U.S. Dollars by 2031 (www.worldseed.org/, www.euroseeds.eu/). To support such an impressive expansion—in line with the current global demand for increased food, feed, and biofuel production—continuous technological innovations should be provided to seed technologists, breeders, and farmers. There is strong pressure on these issues also because climate change is disrupting food security at the global level, a condition that has been further exacerbated by the coronavirus pandemic. The impact of COVID-19 on the production of certified seeds has been assessed by international institutions committed to developing strategies to preserve seed availability, accessibility, and quality (www.fao.org/family-farming/detail/ar/c/1331528/). The adverse effects of COVID-19 include a decreased production of seeds with certified quality due to the limited accessibility to the companies that supply certified seeds, along with challenges related to seed mobilization and field-based inspections (Nchanji et al. 2021).

**Fig. 1:**
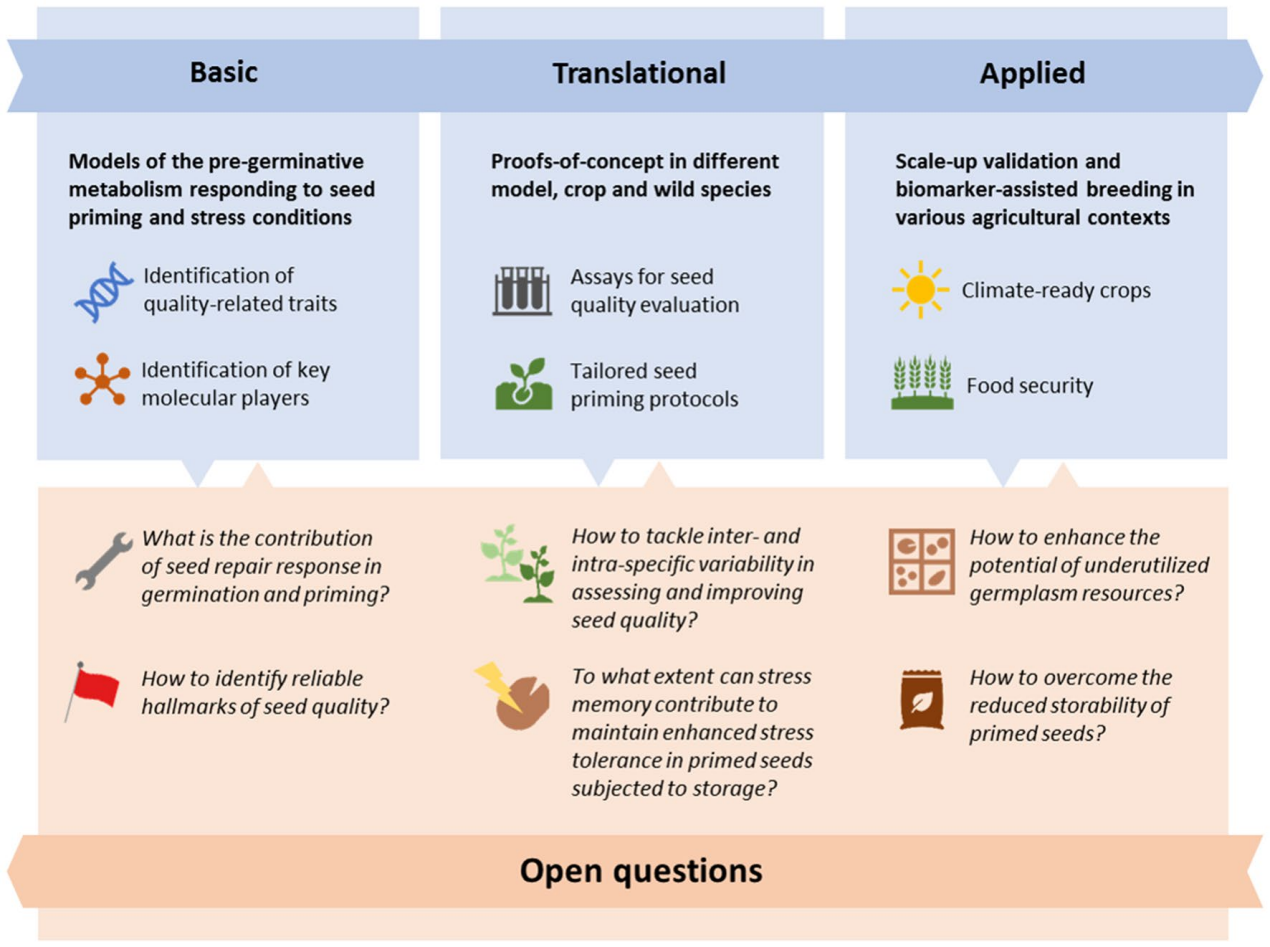
Overview of the implications of seed priming technologies throughout basic, translational, and applied research, highlighting the most relevant deliverables of each phase and stating the main open questions driving future developments

Farmers preferentially use commercial seeds with enhanced quality, resulting from the technological advances achieved by seed companies. High-vigor seeds offer several advantages, including high yields, improved nutritional value (Veena and Puthur 2022; Zrig et al. 2022), stress tolerance, and disease resistance. Additionally, different pre-sowing techniques, comprehensively defined as seed priming, are also applied to increase seed vigor. Within these methodologies, seed imbibition is carried out under controlled conditions, in water, or in solutions containing different types of priming agents, followed by desiccation. A successful seed priming requires that controlled hydration is stopped before the occurrence of radicle protrusion, otherwise, seeds will lose desiccation tolerance and thus viability (Paparella et al. 2015; Lutts et al. 2016). This simple definition describes the ability to act on the seed pre-germinative metabolism and stimulate events that are crucial for seed quality, required not only to enhance germination performance but also to boost seedling stress resilience. In this review, an update of the current scientific and technical knowledge related to seed priming is provided. Emphasis is given to the need to expand the basic knowledge of the molecular mechanisms underlying the seed response to priming. The literature so far available highlights the strong potential of basic research on primed seeds as a source of seed quality hallmarks. Looking at the seed metabolism with integrated high-throughput molecular approaches reveals key players (genes, proteins, metabolites) responsible for the ability to repair cellular damage, scavenge toxic radicals, and preserve genome integrity. The current state-of-the-art resulting from basic research must be expanded and, at the same time, fully exploited and translated into effective tools to improve seed priming technology. As shown in Fig. 1, the investigation of different models of pre-germinative metabolism challenged with optimal and/or suboptimal priming treatments represents a main pillar of basic research, whereas translational research requires proof-of-concept to validate the potential of novel seed quality hallmarks. The workflow outlined in Fig. 1 leads to the final step of applied research that is expected to provide reliable solutions for climate-ready crops and food security. Despite the efforts and encouraging results, the progression of this workflow is hampered by the numerous open questions that are still pending, listed in Fig. 1. All these aspects are discussed in different sections of this review.

### Climate change, seeds, and crop productivity

Changes in global temperatures are dramatically altering the frequency and intensity of weather events, including heat waves, exposure to freezing conditions, drought periods, and precipitations. Extreme weather fluctuations are already affecting crop yields at the global scale. Indeed, the expected increase in agricultural production does not match the urgent need to feed the growing population of our planet, threatening future food security (Thiault et al. 2019; Calleja-Cabrera et al. 2020). The multifaced effects of climate change need to be considered at multiple levels, from seed/plant physiology to the socioeconomic factors involved in different agricultural realities.

#### Direct impact of greenhouse gases

Among the most severe challenges of climate change, the accumulation of greenhouse gases (CO_2_, O_3_, CH_4_) impacts crop physiology at different levels, including the occurrence of oxidative damage, decreased photosynthetic efficiency, and accelerated senescence (Wang et al. 2019). The increasing trends in atmospheric CO_2_ (25% higher since the levels recorded in 1959) have been suggested to alter carbon/nitrogen ratios, with consequences on nitrogen availability (Ziska et al. 2012; Lamichaney and Maity 2021).

#### Heat waves

The rising temperatures triggered by the accumulation of greenhouse gases cause severe effects on plants, mediated by the deterioration of macromolecules and by the exacerbation of oxidative stress. In this context, ROS accumulation associated with heat waves can alter hormonal signaling and the regulation of seed dormancy (Finch-Savage et al. 2017; Zhou et al. 2020; Farooq et al. 2021a, b). From an agricultural standpoint, such effects are particularly evident for cold-adapted crops, such as cool-season grain legumes, namely chickpeas (*Cicer arietinum* L.), lentils (*Lens culinaris* Medik.), and fava beans (*Vicia faba* L.), with substantial losses in terms of yields and nutrient content (Fahad et al. 2017; Kumar et al. 2021).

#### Drought events

Water availability is a major limiting factor for plant development and an economical issue for many farming systems. In plants at the reproductive stage, drought stress results in pollen sterility, ovary abortion, and reduced kernel number/biomass. Drought events associated with climate change are affecting crop yield worldwide, causing irreversible damage (Boyer and Westgate 2004), as yield losses under drought conditions have been estimated for many major crops, including maize (*Zea mays* L., 63–87%), wheat (*Triticum aestivum* L., 57%), rice (*Oryza sativa* L., 53–92%), and legumes, such as chickpea (*Cicer arietinum* L., 45–69%) and soybean (*Glycine max* L., 46–71%) (Fahad et al. 2017).

#### Flooding

Intense rainfall, often associated with floods occurring during key stages of crop growth, compromises yields and harvest quality since both plant biomass and seed size are reduced. Moreover, besides the stress caused by the anoxic conditions associated with prolonged submergence, flooding events occurring in coastal areas expose crops to osmotic and salt stress (Hanley et al. 2019).

Based on the available data on climate dynamics and crop productivity, Ray et al. (2019) used linear regression relationships to assess the impact of climate change on ten major crops, namely barley (*Hordeum vulgare* L.), cassava (*Manihot esculenta* L.), maize, oil palm (*Elaeis guineensis* L.), rapeseed (*Brassica napus* L.), rice, sorghum (*Sorghum bicolor* L.), soybean, sugarcane (*Saccharum officinarum* L.), and wheat. According to this study, the impact of global climate change on crop yields ranged from -13.4% (oil palm) to 3.5% (soybean), with variable geographical distribution. A negative impact was observed in Europe, Southern Africa, and Australia, mixed situations were reported in Asia and Northern and Central America, whereas Latin America was characterized by positive effects. Such a scenario has already led to a reduction in consumable food calories in these crops (Ray et al. 2019), underlining the centrality of seed quality improvement as a necessary strategy to adjust different farming systems in response to climate change. Hence, the advantages of seed priming as a versatile and resource-effective approach for seed quality improvement under different environmental conditions need to be highlighted, understood, and applied.

### Priming technology: from traditional to innovative methods

#### From Theophrastus to nanoparticles: a time travel

In his pivotal writings “History of Plants” and “Causes of Plants”, Theophrastus of Eressus (371–287 B.C.), explored different aspects of seed biology, from seed production to germination and conservation (Evenari 1980, 1984; Thanos 1994, 2007), observing that cucumber (*Cucumis sativus* L.) seeds soaked in milk or water before sowing resulted in faster germination (Theophrastus, Enquiry into Plants, Book VII, I.6). The Roman naturalist Gaius Plinius Secundus (Pliny the Elder, A.D. 23–79) underlined in his Naturalis Historia the relevance of presoaking seeds to improve germination (Evenari 1984). The French agronomist and botanist Oliver de Serres (1539–1619) described the effectiveness of the treatment used by farmers on grains (*Triticum*, *Secale,* and *Hordeum* spp.) in which seeds were soaked for 2 days in manure water and then dried in the shade before sowing. Even Charles Darwin (1809–1882) conducted several experiments on seed germination, resulting in observations that contributed to his theory on the evolution of living organisms (Black 2009). He tested osmopriming by submerging in salty sea water the seeds from different plant species. The treatment was able to enhance germination for some of the tested seeds (Darwin 1857). The seed ability to survive in salt water suggested to Darwin that long-distance dispersal was a possible explanation for the geographical distribution of species (Black 2009).

Results from subsequent studies available at the beginning of the nineteenth century contributed to formulating the modern concept of seed priming. Kidd and West (1918, 1919) initially defined the dangerous practice of soaking dwarf bean (*Phaseolus vulgaris* L.) and pea (*Pisum sativum* L.) seeds before sowing. They also underlined that, despite this evidence, gardeners were still soaking their pea and bean seeds "to help them", taking advantage of their empirical ability to adjust the proper amount of water (Kidd and West 1918, 1919). The transition from this empirical awareness to a well-defined concept of seed priming, as a reproducible technique at the service of people working with seeds, started in the 1960s, with the early work of May et al. (1962) who evidenced that treated seeds dried under controlled conditions displayed fast germination under physiological and stress conditions. Then Ellis (1963) reported that the emergence of tomato (*Solanum lycopersicum* L.) seedlings was improved when seeds were treated with solutions containing different salts (K_3_PO_4_, KNO_3_, NaCl). Heydecker and his coworkers imbibed the seeds of several horticultural and ornamental crops using a solution of the osmotic agent polyethylene glycol (PEG), obtaining accelerated and uniform germination (Heydecker et al. 1973). The impact of the treatments was analyzed based on osmotic potential, temperature, duration, and the efficacy of their combinations. The successful output prompted the authors to comment on the “fascinating physiological implications” of this technique (Heydecker et al. 1973), subsequently named the “priming” of seeds (Heydecker and Gibbins 1978).

Since then, the use of seed priming has progressively increased, and different types of protocols have been developed. Among them, hydro-, osmo-, hormo-, chemo-, and bio-priming are widely applied and declined in many variants, tailored on plant genotype and seed lot, envisaged as tools to fight the current challenges of agriculture and ecosystems (Paparella et al. 2015; Marthandan et al. 2020; Paul et al. 2021). Our time travel continues nowadays with the noteworthy technological revolution brought by nanotechnology in agricultural research, in terms of seed quality enhancement and the ongoing discussion on the sustainability of nanomaterials (Chandrasekaran et al. 2020; Shelar et al. 2021; Amritha et al. 2021, do Espirito Santo Pereira et al. 2021).

#### Main features and priming treatments

**Fig. 2:**
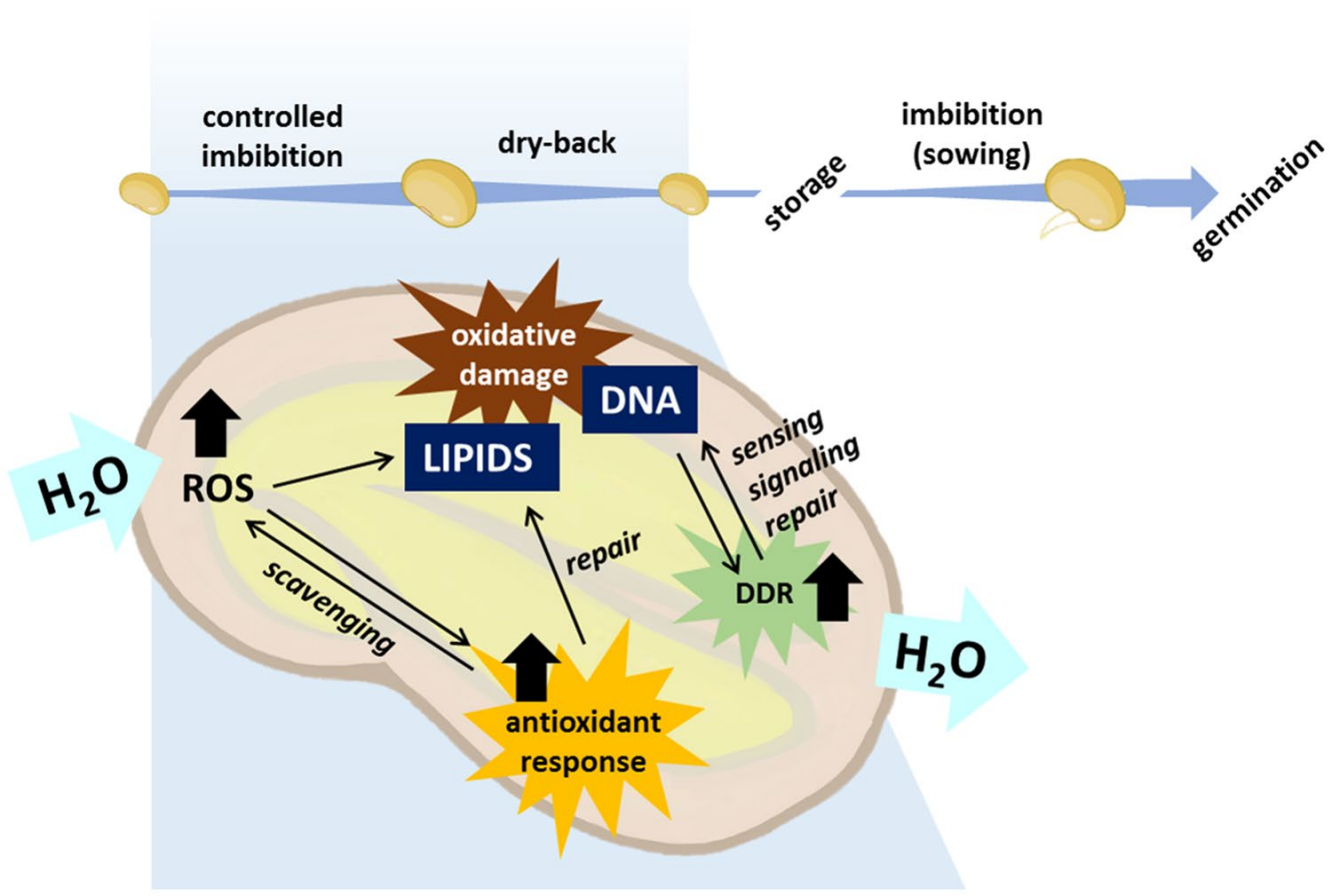
The rehydration–dehydration cycle as a schematic representation of the treatment (controlled seed imbibition followed by desiccation or dry-back) routinely applied in standard seed vigorization protocols. Primed seeds undergo post-priming germination. The different steps of the rehydration–dehydration cycle can be regarded as potential sources of novel seed quality hallmarks, specified as genes, proteins, metabolites

When seed priming is applied, controlled water uptake allows a boost of seed metabolism and accelerates germination, but it is mandatory to avoid the reach of the radicle protrusion stage (Soeda et al. 2005). If the seeds exceed this critical threshold, the germination process becomes irreversible, desiccation tolerance is lost and the dry‐back step will result in the death of these desiccation-sensitive seeds (Bradford et al. 1990). The concept of rehydration–dehydration cycle (Fig. 2) is used to represent the treatment (controlled seed imbibition followed by desiccation or dry-back) routinely applied in standard seed vigorization protocols. Such a concept represents an ideal biological context to explore some critical issues of the technique, related to loss and gain of desiccation tolerance (Fabrissin et al. 2021; Farooq et al. 2019; Paparella et al. 2015; Pagano et al. 2022a, 2022b). Some of the main conventional seed priming techniques (hydropriming, solid matrix priming, osmopriming, chemopriming, hormopriming, thermopriming) are briefly described in this paragraph along with hybrid methodologies and the most recent applications based on nanomaterials.

#### Hydropriming

This technique represents a cost-effective, and eco-friendly priming approach that requires soaking seeds in water only and the subsequent dry-back to their original weight. Each crop genotype shows a distinctive critical time threshold for soaking, below a safe limit that must be analytically assessed to gain the best impact on seed vigor (Harris et al. 2001). The treatment improves germination performance, seedling emergence, crop yield, and stress resilience (Farooq et al. 2010; Damalas et al. 2019; Khan et al. 2020a). Hydropriming has been tested in several climate-sensitive regions (Harris et al. 2001; Binang et al. 2012; Matsushima et al. 2013; Nakao et al. 2018, 2020; Adhikari et al. 2021). Yield improvement reached 28% in dicot plants, especially legumes, and 15% in cereals, respectively. However, treatments turned out to be more advantageous under abiotic stress conditions (22–28% yield enhancement) (Carrillo-Reche et al. 2018). Uncontrolled water uptake by the seeds represents the weakness point of hydropriming, since imbibition depends on the affinity of the seed tissues to water. For this reason, it is crucial to understand the optimal water volume, temperature conditions, and duration of the treatment to prevent radicle protrusion and the subsequent loss of desiccation tolerance. Despite the evident need to optimize protocols on a genotype scale or even seed lot scale, hydropriming remains the preferred option for seed technologists, breeders, and farmers, particularly when facing harsh agro-climatic conditions.

#### Solid matrix priming (SMP)

Seeds are mixed with a solid carrier that is water moistened to establish the desired water potential for effective priming, in such a way that imbibition is slowed down resembling the natural rehydration process in the soil. After the treatment, the solid matrix material needs to be mechanically separated without damaging the seeds (Damalas et al. 2019). Different types of solid insoluble matrices are available, e.g., charcoal, Cocopeat, Sphagnum moss, perlite, vermiculite, Celie or Micro Cell, diatomaceous earth clay, and sand. They can be combined to strengthen the priming effect as reported by Madsen et al. (2018), who tested a mixture containing calcium bentonite clay, diatomaceous earth, compost, worm castings, non-ionic alkyl terminated block copolymer surfactant, a plant growth regulator, a fungicide, a liquid fertilizer, and tap water. SMP carried out under enriched O_2_ conditions turned out to be effective on low-quality leek (*Allium ampeloprasum* L.) seeds (Ozden et al. 2018). SMP improves the pre-germinative metabolism, field emergence, and establishment, especially in horticultural crops (Grzesik and Nowak 1998; Lutts et al. 2016), cereals (Hacisalihoglu et al. 2018), and native grasses (Madsen et al. 2018). SMP provides the advantage of exerting a more effective control on moisture content and water potential during seed imbibition. However, some upgrades and optimization are still needed. A significant improvement in this technique has been achieved by developing novel and sustainable procedures that do not require separating seeds from the matrix material (Madsen et al. 2018).

#### Osmopriming

Seeds are subjected to osmopriming using aerated solutions containing potassium nitrate (KNO_3_), potassium phosphate (K_3_PO_4_), potassium chloride (KCl) salts, NaCl, CaCl_2_, MgSO_4,_ or polyethylene glycol (PEG) with different water potentials, applied for different periods (Biswas et al. 2019; Feghhenabi et al. 2020; Lei et al. 2021, Hussain et al. 2022). In presence of osmotic agents, the amount of water entering the seeds is limited but sufficient to trigger the pre-germinative metabolism. In some cases, germination rate and percentage are more responsive to soaking time rather than water potential imposed by the osmotic agent (Abdallah et al. 2016; Mirmazloum et al. 2020). Salt priming enhances tolerance to salinity (Janda et al. 2016), heat (Qiu et al. 2003), and chilling (Cheng et al. 2014). PEG-primed seeds show improved germination and seedling growth rates, as well as stand establishment under drought (Goswami et al. 2013; Zhang et al. 2015), temperature stress (Murray et al. 1993; Parera and Cantliffe 1992; Bush et al. 2000; Nascimento et al. 2013; Lei et al. 2021, Hussain et al. 2022), or in presence of multiple stresses (Bittencourt et al. 2004; Chen et al. 2010). PEG is generally described as a successful priming agent (Moosavi et al. 2009; Patanè et al. 2009; Pradhan et al. 2015; Patade et al. 2015; Moreno et al. 2017; Abid et al. 2018; Hussain et al. 2019a; Nadeem et al. 2019), with some exceptions (Shahi-Gharahlar et al. 2009). Despite the huge number of reports describing the molecular and physiological mechanisms underlying the seed response to osmopriming under specific stress conditions, the current knowledge concerning the potential of osmopriming to strengthen the response against multiple stress factors is still scanty (Lei et al. 2021).

#### Halopriming

Priming with salt solutions is also referred to as halopriming. Seeds are soaked in solutions containing inorganic salts, e.g., NaCl, KNO_3_, CaCl_2_, CaSO_4_. Halopriming with NaCl was reported to enhance germination and seedling establishment in milk thistle (*Silybum marianum* L., Sedghi et al. 2010), as well as to increase salt tolerance of melon (*Cucumis melo* L., Sivritepe et al. 2005), canola (*Brassica napus* L., Farhoudi et al. 2007), sugarcane (Patade et al. 2009), and *Vigna radiata* L. (Jisha and Puthur 2014a). Halopriming applied to rice seeds resulted in enhanced protein, carbohydrate, and photosynthetic pigment content, along with antioxidant enzyme activities associated with reduced lipid peroxidation levels under salt stress (Jisha and Puthur 2014b). Halopriming treatments of maize seeds with NaCl improved germination and seedling biomass, leading to higher grain yield and water use efficiency in the field, particularly under drought stress conditions (El-Sanatawy et al. 2021). Halopriming applied to seeds triggers plant stress memory, preventing the deleterious impact of abiotic stresses such as drought and salinity (El-Sanatawy et al. 2021). This intriguing issue, currently under investigation, might provide novel insights into the molecular networks of seed priming and maximize the hidden potential of this technique (Srivastava et al. 2021).

#### Chemopriming

Exogenous and plant-derived chemicals are used as priming agents. An increasing range of molecules playing a signaling role in the plant stress response can act as priming agents. The list includes H_2_S, NO, and natural compounds (e.g., chitosan, melatonin, ascorbic acid, alpha-tocopherol, trehalose, and polyamines), and plant extracts. Their efficacy to enhance salt tolerance has been reported in different crop species, and mechanisms underlying the impact of these priming agents on the seed pre-germinative metabolism are also documented (Zulfiqar et al. 2022). Silicon-mediated seed priming gained attention as a strategy to induce stress adaptation (Farooq et al. 2021a; El-Serafy et al. 2021). The range of potential new agents for chemopriming is expanding, as reported for sodium nitroprusside (Hameed et al. 2021), 2,6 dichloro-isonicotinic acid (Martinez-Aguilar et al. 2021), pineapple stem-derived protease (Perez et al. 2021), polyamines and humic acid (Sheteiwy et al. 2017; Mridha et al. 2021; Hongna et al. 2021). Plant extracts containing bioactive molecules with high antioxidant potential can be effective priming agents, as reported for rice seeds treated with a carrot root extract rich in carotenoids, phenolic compounds, tocopherols, nitrogen compounds, and vitamins (Bigolin Teixeira et al. 2021). Chemopriming will benefit from the expanding technology for waste recycling since the valorization of by-products (e.g., from the processing of fruits, vegetables, tubers, cereals, and legumes) is expected to provide novel ingredients for sustainable seed priming formulations.

#### ROS-mediated priming

The beneficial effects of exogenous hydrogen peroxide (H_2_O_2_) have been documented (Wahid et al. 2007; Khan et al. 2015; Wojtyla et al. 2016; Dufková et al. 2019). H_2_O_2_ promotes germination within a proper dose range whereas it becomes toxic at high levels (Bailly et al. 2008). H_2_O_2_ is a signal molecule involved in phytohormone metabolism, such as SA-mediated signaling pathways. The synergistic action of H_2_O_2_ and SA was able to boost germination in maize seeds by promoting the antioxidant response and energy metabolism, particularly under chilling (Luo et al. 2017) and in kidney beans subjected to salt stress (Tania et al. 2022). A similar protective role of H_2_O_2_-mediated priming was highlighted in rice seeds and seedlings under drought stress in rice (Jira-Anunkul and Pattanagul 2020), as well as cauliflower seeds and seedlings challenged with salt stress (Ellouzi et al. 2021). Furthermore, H_2_O_2_ could improve the photosynthetic efficiency in sunflower plants developed from primed seeds, under salt stress (Silva et al. 2022). The complex cellular and molecular networks in which H_2_O_2_ is an active player cover a range of developmental and stress responses that require cross-talk with phytohormones and antioxidant mechanisms, sometimes ruled by genetic features. Such complexity should be more extensively investigated to provide knowledge useful to optimize the H_2_O_2_-based treatments.

#### Hormopriming

The use of different phytohormones as priming agents has become an established approach with proven beneficial effects in promoting plant stress tolerance (Rhaman et al. 2021). Successful seed priming has been reported using auxin (indole-acetic acid, IAA) (Eisvand et al. 2010; Fahad et al. 2015), cytokinins (CKs) (Bryksova et al. 2020), gibberellins (GAs) (Ghobadi et al. 2012; Ma et al. 2018), and abscisic acid (ABA) (Gurmani et al. 2011, 2013; Wei et al. 2015; Zongshuai et al. 2017; Safari et al. 2018). Seed priming with ethylene-related compounds was applied to lettuce (*Lactuca sativa* L.) to increase germination under high temperatures (Nascimento et al. 2004). Successful salicylic acid (SA)-mediated priming was also reported (Rehman et al. 2011; Li et al. 2017a; Khan et al. 2019; Karalija et al. 2021; Zhu et al. 2021). Jasmonic acid (JA) has been used as a priming agent to treat tomato seeds, enhancing the seedling ability to withstand nematode attacks (Bali et al. 2020). Priming with brassinosteroids (BRs) significantly enhanced tolerance to heavy metal toxicity (Basit et al. 2021, 2022), and drought (Huang et al. 2020), whereas the use of melatonin contributed to improved cold tolerance (Cao et al. 2019; Kołodziejczyk et al. 2021). Due to the complexity of phytohormone metabolism and regulatory networks, the selection of suitable conditions and effective molecules for hormopriming depends on a deeper knowledge of several key molecular players and the way they could contribute to boosting germination (Bryksova et al. 2020).

#### Thermopriming

Heat priming can be applied to hydrating or germinating seeds that are exposed to low or high temperatures. Heat priming provided advantages to *Arabidopsis* (Serrano et al. 2019) and *Triticum aestivum* L. (Fan et al. 2018) exposed to high temperatures. The stimulating impact of raising temperature on bread wheat seed germination was reported by Gerna et al. (2018), who showed how a commercial hot steam treatment was able to advance seed metabolism and redox shifts associated with germination and seedling growth. Ahmad et al. (2020) reported that thermopriming applied to *V. radiata* L. seeds, by treating at 4 °C for about 1 h followed by drying, had a significant impact on chlorophyll, carotenoids, protein, and proline content. Thermopriming at 60 °C for 6 and 10 h was successfully applied to safflower (*Carthamus tinctorius* L.) seeds resulting in improved yield and percentage of seed oil in the field (Barazandeh et al. 2019). Lentil (*Lens culinaris* Medik.) seeds that underwent heat priming for 6 h at 35 °C displayed beneficial effects, including synthesis of osmolytes and increased photosynthetic performance, particularly evident in heat-sensitive genotypes (Bhardwaj et al. 2021). The potential of thermopriming should be better exploited given the current drawbacks of global warming that affect crop productivity. Mechanisms that link heat priming at the seed level with the plant ability to withstand heat stress in the field should be better clarified.

#### Biostimulants

Complex mixtures derived from raw materials, e.g., waste from food and paper industries, safe for the environment and possessing a broad spectrum of biological activities, are used as biostimulants to improve seed germination (Bulgari et al. 2019; Gupta et al. 2022) and promote the plant defense response (Alzahrani and Rady 2019). Biostimulants based on protein hydrolysates, natural products derived from agricultural waste, can help reduce the use of chemical fertilizers. Given the complexity and heterogeneity of the starting materials, high-throughput automated phenotyping is used to speed up the selection of the best-performing formulations (Sorrentino et al. 2021). Biostimulants restored the oxidative balance in cucumber seeds exposed to heat stress, triggering the expression of *ICL* gene coding for isocitrate lyase, a key enzyme in seed germination (Campobenedetto et al. 2020). Flavonoids extracted from citrus fruits combined with a cell-free supernatant from a novel bacteria *Devosia* sp. SL-43 proved to be an effective priming agent (flavopriming) when applied to soybean and canola seeds under salt stress (Shah et al. 2022). Biostimulants represent a promising avenue in the context of sustainable agriculture, not only for their broad range of beneficial effects on germination and stress tolerance but also for their potential in the implementation of circular economy policies.

#### Nanopriming

Nanoparticles (NPs) of metal oxides, widely used in industries, appear as emerging tools for seed priming purposes in agriculture. The application of selenium and zinc oxide nanoparticles (SeNPs, ZnONPs) during *B. napus* seed imbibition under salinity stress showed how nanopriming was able to modulate the expression of ABA-related genes (El-Badri et al. 2021). Seed priming with titanium dioxide nanoparticles (TiO_2_-NPs) resulted in beneficial effects on biochemical, morphological, and physiological characteristics of coriander (*Coriandrum sativum* L.) plants under Cd stress (Sardar et al. 2022). MgO-based NPs promoted seed germination, growth, and photosynthetic efficiency of maize (Shinde et al. 2020), and *V. radiata* (Anand et al. 2020) seedlings, similar to what was reported for Ca-based NPs in rice (Yugandhar and Savithramma 2013). ZnO-based NPs applied to rice (Prerna et al. 2019) and wheat (Nadeem et al. 2019; Rai-Kalal and Jajoo 2021) could improve productivity. According to Li et al. (2021), ZnO NPs could mitigate Cd toxicity in rice by promoting an increase in seedling weight associated with changes in antioxidant response and metabolic pathways related to DNA/RNA synthesis. Similarly, Salam et al. (2022) reported a higher content of nutrients and antioxidant enzymes in ZnO NPs-primed maize seeds and seedlings able to withstand Co toxicity, with enhanced growth and yield. Promising results were reported when ZnO-based NPs were used to increase seed germination in lettuce (Rawashdeh et al. 2020). NPs containing SiO_2_ used as seed priming agents improved drought tolerance in wheat (Rai-Kalal et al. 2021). Nanoscale micronutrient iron (α-Fe_2_O_3_), prepared via co-precipitation with the marine macroalga *Turbinaria ornata* and used as a priming agent, could enhance seed germination in rice and maize (Prerna et al. 2021). Si-based NPs might help increase plants' biomass and yield while reducing oxidative stress and Cd uptake in wheat grains (Hussain et al. 2019b). Seed priming with commercially available silver nanoparticles (AgNPs) enhanced salinity tolerance in pearl millet (*Pennisetum glaucum* L.) (Khan et al. 2020b). AgNPs-mediated seed priming in Chinese cabbage (*Brassica rapa* subsp. Pekinensis) increased crop yield and nutritional quality with the added value of biosafety, as Ag did not bioaccumulate in edible tissues (Zhou et al. 2021). Nanoparticulate systems represent a sustainable approach to convey bioactive compounds for agricultural applications, as in the case of successful seed priming mediated by alginate/chitosan (nanoALG/CS) and chitosan/tripolyphosphate (nanoCS/TPP) containing GA_3_ performed in tomato (do Espírito Santo Pereira et al. 2019). Multi-walled carbon nanotubes (MWCNTs) were used to prime wheat seeds, resulting in accelerated germination, enhanced growth, and higher yield, providing a new opportunity based on cost-effective nanomaterials for boosting crop performance (Joshi et al. 2018). Despite the recent advances, there is still a gap of knowledge concerning the way nanoparticles might affect seed physiology and microenvironments, e.g., it has been suggested that dry-back can alter the properties of nanomaterials and seed viability (Shelar et al. 2021). For this reason, additional studies are required to mitigate such risk.

#### ‘Green’ priming

Synthesis of plant-based nanoparticles is a further refinement of nanotechnology that uses sustainable manufacturing processes to produce safe and innocuous nanoscale biomaterials for agricultural applications (Singh et al. 2018; Amritha et al. 2021). AgNPs synthesis by laser ablation, irradiation, thermal treatment, or chemical reduction shows several drawbacks, such as energy requirement and use of organic solvents, resulting in hazardous wastes and difficulty in scale-up. Differently, green synthesis is low cost, safe, and eco-friendly since nanoparticles contain organic materials (lipids, proteins, polysaccharides) with unique physical and chemical properties (Masukar et al. 2011, Mochochoko et al. 2013). Ag-based ‘green’ nanoparticles have an added protective advantage because of Ag anti-bactericidal and anti-fungicidal properties. Polysaccharides extracted from *Chlorella vulgaris* were used to produce AgNPs showing antimicrobial activity against *Bacillus* sp., *Erwinia* sp., *Candida* sp., and effective seed priming in *Triticum vulgare* and *P. vulgaris* (El-Naggar et al. 2020). Turmeric oil nanoemulsions (TNE) and AgNPs synthesized from agro-industrial byproducts (curcumin-removed turmeric oleoresin combined with onion (*Allium cepa* L.) peel extract as reducing agent) were used as nanopriming agents for watermelon (*Citrullus lanatus*) seeds. This eco-friendly and sustainable nanotechnological approach enhanced seed germination, growth, and yield while maintaining fruit quality (Acharya et al. 2020). Biocompatible FeO NPs synthesized using *Cassia occidentalis* L. flower extracts were tested as nanopriming agents and shown to promote germination of Pusa basmati rice seeds, by enhancing α-amylase activity, iron acquisition, and elevated soluble sugar levels (Afzal et al. 2021). The effects of nanopriming with galactomannan-stabilized phyto-complexed calcium hydroxide (Ca(OH)_2_), selenium oxyanion-calcium hydroxide SeO-(Ca(OH)_2_), and selenium-calcium hydroxide Se-(Ca(OH)_2_) nanocomposites were tested in *V. radiata* seeds. Seed extracts from *Cassia angustifolia*, rich in galactomannan and other biomolecules, enabled their terminal oxygen and hydroxide groups to bind Ca and Se ions. The porous Se-(Ca(OH)_2_) nanocomposite showed high efficacy in interacting with seed embryos and stimulating germination (Antony et al. 2022). Nano-scale zero-valent iron (G-nZVI), synthesized using fruit peel waste of *Punica granatum* L., could increase the germination percentage and seedling vigour of rice (Guha et al. 2021). Nanostructured lignin microparticles (LNP), obtained from alkaline lignin by acid treatment, were tested on maize seeds resulting in beneficial effects (Del Buono et al. 2021). ZnO NPs obtained through green synthesis using *Senna occidentalis* leaf extract were used to prime-aged Pusa basmati rice seeds (Sharma et al. 2021). With ‘green’ priming, the opportunity to meet higher and better yields with sustainability is further enhanced, while the continuous advances in the field of nanotechnology will contribute to fully exploiting the potential of this technique.

#### Hybrid priming

Multiple priming agents are combined and used synergically to boost multiple levels of stress tolerance in crop plants. Hydro-electro hybrid priming (HEHP) was successfully applied to onion (Zhao et al. 2018) and tomato (Garcia et al. 2021) seeds by combining hydropriming with exposure to electrostatic field irradiation. Positive outputs were also reported when combining hormopriming with osmopriming in broccoli (Hassini et al. 2017) and with hydropriming in sunflower (Górnik and Lahuta 2017). GA and H_2_O_2_ were combined to treat tobacco (*Nicotiana tabacum* L.) seeds using diphenylene iodonium chloride and uniconazole, as inhibitors of H_2_O_2_ and GA synthesis, respectively. The study evidenced that GA and H_2_O_2_ were essential for seed germination by decreasing ABA/GA ratio and stimulating reserve mobilization (Li et al. 2018). Priming *Sulla carnosa* seeds with SA and H_2_O_2_ significantly improved the growth performance and the rhizosphere acidification of iron-deficient plants (Jelali et al. 2021). Benefits were observed with the combined application of phytohormones and other growth regulators (Sharma et al. 2020), as in the case of lentil seeds exposed to γ-aminobutyric acid (GABA) and heat priming (Bhardwaj et al. 2021). The diverseness of seed priming protocols provides the opportunity to combine different approaches in the attempt to generate a synergic positive impact on the seed pre-germinative metabolism. Possibly, dealing with richer assortments of priming agents will require more complex and time-consuming procedures, although this challenge would result in targeted and sustainable hybrid priming protocols.

### Pre-germinative metabolism: the key to understanding seed priming

To provide an overall description of the germination process while also taking into account inter-specific variability, Bewley (1997) proposed a triphasic pattern based on the dynamics of water uptake. At the onset of the germination process (Phase I), seed coat permeability and tissue capillarity physically drive the water uptake required for the resumption of seed metabolism. During the subsequent phase (Phase II), the seed water potential is balanced, leading to a reduction in the rate of water uptake as metabolism transitions toward germination. With post-germination (Phase III), further water uptake is associated with radicle protrusion, with the consequent increase in water content associated with seedling establishment. From a metabolic perspective, the transition from imbibition to radicle emergence implies the consequential activation of energy metabolism, DNA and membrane repair mechanisms, turnover of stored transcripts, de novo transcription and translation, cell elongation, and reservoir mobilization (Bewley and Black 1994; Bewley 1997; Nonogaki et al. 2010).

**Fig. 3:**
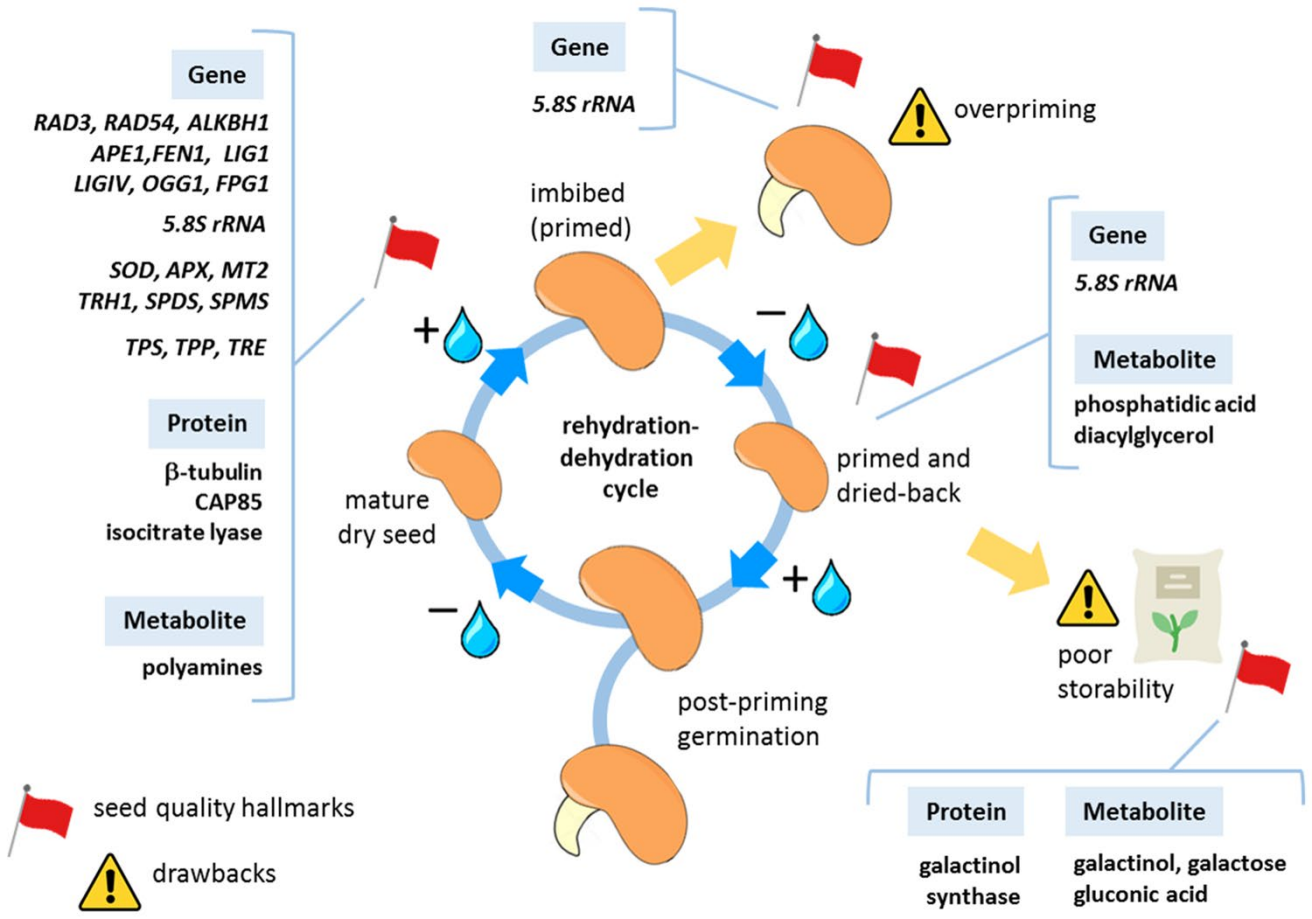
Molecular mechanisms of seed priming. The main drawbacks of the technique, overpriming and reduced shelf-life of primed seeds are also represented

**Fig. 4:**
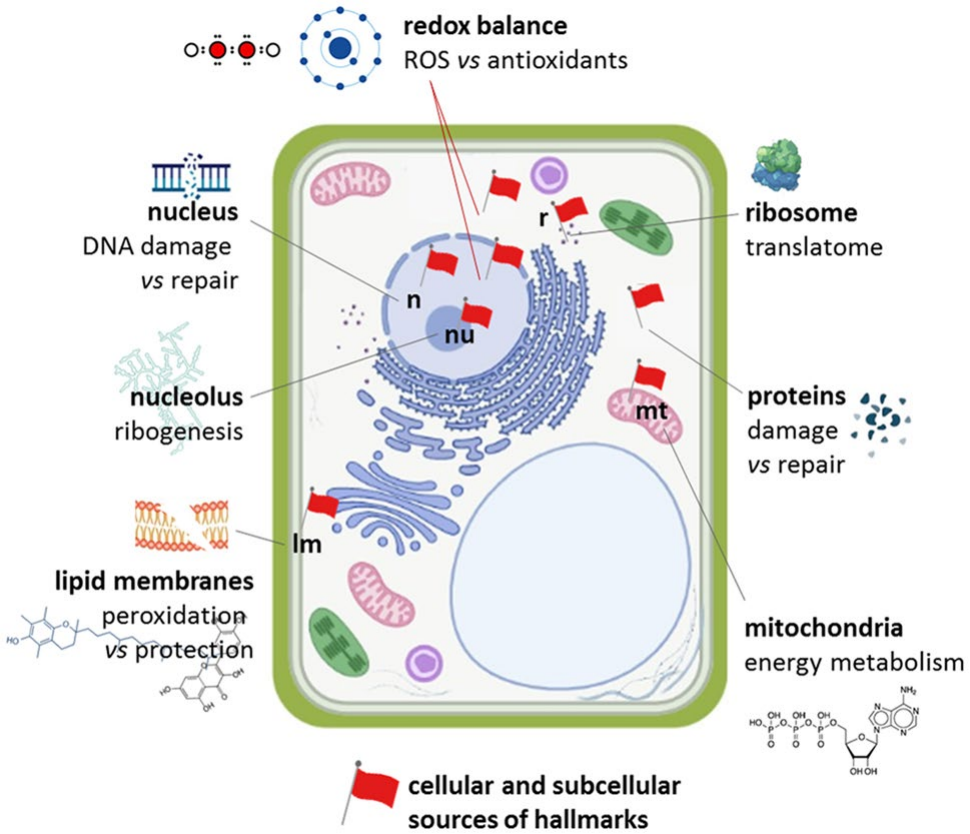
Representation of the cellular and subcellular sources of the different seed quality hallmarks currently under investigation. *ROS* reactive oxygen species, *n* nucleus, *nu* nucleolus, *mt* mitochondria, *lm* lipid membrane, *r* ribosome

The first wave of metabolic activities triggers energy production and reserve remobilization, the translation mechanism, and protects against oxidative stress by promoting antioxidant and repair pathways (Rajjou et al. 2012; Domergue et al. 2019). This highly complex and sophisticated plethora of molecular and metabolic pathways confined within the early germination temporal frame has been termed ‘pre-germinative metabolism’ (Paparella et al. 2015; Macovei et al. 2017). To date the scientific literature has provided increasing evidence of the role played by different components of the pre-germinative metabolism in the seed response to priming, strengthening the idea that a deeper understanding of such mechanisms represents a fundamental path toward “next-generation” priming tools. The main pathways contributing to the seed response to priming, and currently regarded as potential sources of novel seed quality molecular hallmarks, are described below whereas a graphical representation of their cellular and subcellular localization is provided in Fig. 3 and Fig. 4, respectively.

#### Energy metabolism as an indicator to assess the seed's physiological response to priming

Respiration is a key process during early seed imbibition, dedicated to fulfilling the energy requirements of the developing heterotrophic embryo (Bewley 1997). ATP synthesis and oxygen consumption occur rapidly upon rehydration, and this requires the presence of intact and functional mitochondria in the desiccated embryo cells (Raveneau et al. 2017; Paszkiewicz et al. 2017; Nietzel et al. 2020), although other reports underlined the need for maturation of protomitochondria before respiration can start (Law et al. 2012). In a recent study, Nietzel et al. (2020) were able to define the kinetics of reactivation of mitochondrial energy and identify the target cysteine residues acting as thiol switches in early germination, highlighting the impact of redox metabolism and its link to energy-related pathways. Depending on both seed type and available substrates, the respiration activity significantly differs. Raymond et al. (1985) carried out a comparative study on starchy and fatty seeds, evidencing that under aeration ATP was predominantly generated by respiration in all the tested seeds whereas fermentation significantly contributed only in some species. Under anoxia, starchy seeds (e.g., peas and maize) performed better in terms of energy metabolism than fatty seeds (e.g., lettuce) (Raymond et al. 1985). The fermentative pathway is less effective in ATP production and excess accumulation of the related products (e.g., acetaldehyde and ethanol) compromises seed germination (Zhang et al. 1995). ATP is required to support high germination rates (Ching 1973; Lunn and Madsen 1981; Kibinza et al. 2006; He et al. 2019; Qu et al. 2020), to help seeds withstand deterioration (Anderson 1977), and to accomplish successful seed priming (Corbineau et al. 2000) although reports are underlining how high ATP content (Perl 1986; Corbineau et al. 2002), as well as extracellular ATP levels (Wang et al. 2022), can affect germination. Despite contrasting reports, energy metabolism—measured, e.g., in terms of energy charge and ATP:ADP ratio—could be an informative target to explore the impact of priming treatments on seed physiology (Corbineau et al. 2002; Marthandan et al. 2020) (Fig. 3).

#### The seed translatome provides insights into the mechanisms ruling germination and priming

Translatome (the global picture of ribosome-associated mRNA) has been recently used to assess changes in protein levels during seed germination (Bai et al. 2021). By looking at the translatome, it is possible to capture and integrate those multiple and variable regulatory events that modulate protein synthesis and define cell physiology. Besides the traditional approach of polysomal profiling, more recent technological advances allow to define the position of ribosomes at the level of codons (King and Gerber 2016), thus providing a closer perspective on the translatome dynamics. Messenger RNAs accumulated during seed maturation are translated during early seed imbibition (Rajjou et al. 2004). Selected stored mRNAs, loaded into polysomes, include those involved in redox processes, glycolysis, and protein synthesis (Sano et al. 2020). The comparison between proteome, transcriptome, and polysome changes, along with the different germination phases, has allowed to integrate multi-level data and gain novel insights into the translational control of seed germination. This can be envisaged either as a strategy evolved to limit energy consumption or as a sort of checkpoint for germination to occur (Bai et al. 2021). The direct recruitment of ribosomal subunits on *cis*-acting elements (internal ribosome entry sites-IRES), mediated by IRES-specific *trans*-acting factors (ITAFs), is a key step that triggers m7G cap-independent translation of mRNAs investigated in the context of seed germination (Sano et al. 2020). One of these ITAF players, the ErbB3-binding protein (EBP1), was found to over-accumulate in primed sugarbeet (*Beta vulgaris* L.) seeds (Catusse et al. 2011). De novo protein synthesis resulted in the significant production of antioxidant enzymes involved in ROS (reactive oxygen species) scavenging (Galland et al. 2014), driving attention to the complex role of ROS in seed germination and priming. The seed translatome, a crucial element to understand the complex regulatory pathways of germination, can be also regarded as a potential source of seed quality hallmarks (Fig. 3 and Fig. 4), respectively.

#### The seed antioxidant response and its impact on successful priming

The dual role of ROS in early seed germination has been extensively dissected (Bailly et al. 2004, 2008; Kranner et al. 2010; Jeevan Kumar et al. 2015; Bailly 2019). ROS generated during the rehydration of seeds represents a major source of cellular damage (Kibinza et al. 2006; Kurek et al. 2019). On the other hand, successful germination relies on the well-known ‘oxidative window’, in which ROS act as signal molecules and trigger germination (Oracz et al. 2007, 2016, Wojtyla et al. 2016; Barba-Espin et al. 2011; Bailly 2019). The oxidative window is established thanks to the essential and tightly regulated ROS-scavenging activity of both enzymatic and non-enzymatic players (Bailly et al. 2008; Bailly 2019; Bailly and Merendino 2021; Li et al. 2022a). Besides ROS, also RNS (reactive nitrogen species) perform as signaling molecules to promote germination within the oxidative window (Jeevan Kumar et al. 2021; Farooq et al. 2021b). Overall, the connection between a high antioxidant performance observed in the pre-germinative metabolism window and the seed's ability to display improved germination and seedling growth under environmental stresses is consolidated by an increasing body of literature. Such a correlation has become a reference point for the validation of novel formulations that combine conventional and innovative priming agents applied to a wide range of crop varieties. Bioregulators, e.g., auxins, gibberellins, cytokinins, abscisic acid, brassinosteroids, polyamines, strigolactones, and ascorbic acid, provide effective protection against oxidative stress due to their ability to modulate the plant antioxidant system. Given this relevant role, they can be applied as priming agents alone, or in combination with other treatments (Zulfiqar and Ashraf 2021). The dynamics of ROS accumulation versus the seed antioxidant response are envisaged as sources of potential seed quality hallmarks (Fig. 3). The impact of different seed priming techniques has been evaluated in terms of antioxidant response in cereals (Hameed et al. 2019; Cao et al. 2019; Sen and Puthur 2020; Khan et al. 2020a; Shah et al. 2021; Guo et al. 2022), legumes (Kesharvarz et al. 2017; Lilya et al. 2019; Forti et al. 2020b; Chen et al. 2021; Pagano et al. 2022a, 2022b), Solanaceae (Anand et al. 2019; Ali et al. 2019; Bali et al. 2020; Forti et al. 2020a, 2021), and tree species (Zhai et al. 2022). The beneficial effects of seed priming can extend beyond germination and seedling development since these treatments contribute to enhancing the antioxidant defense in field-grown plants (Fig. 4). Seed priming with ascorbic acid could enhance salt tolerance, boosting the antioxidant response of tomato Micro-Tom plants and improving growth and fruit yield (Alves et al. 2021). Furthermore, the enhanced antioxidant activity promoted by seed priming contributes to plant recovery from stress (Aswathi et al. 2022).

#### DNA damage response: the key to preserving genome integrity in seeds

A key aspect of the pre-germinative metabolism is the molecular network underlying DNA damage sensing, signaling, and repair, namely the highly conserved DNA damage response (DDR), essential for genome maintenance (Bray and West 2005; Nikitaki et al. 2018; Waterworth et al. 2010, 2015, 2016, 2019, 2022; Kiran et al. 2020). The entire set of the genetic information stored inside the DNA double helix must be preserved by removing any deleterious change resulting in oxidative stress induced by either exogenous factors or endogenous by-products accumulated along the seed life cycle (Jeevan Kumar et al. 2015; Waterworth et al. 2019; Kurek et al. 2019). Pioneering work has set the starting scenario for disclosing the issues of DNA repair in germinating seeds (Cheah and Osborne 1978, Osborne 1980, 1983; Zlatanova et al. 1987; Dandoy et al. 1987; Elder and Osborne 1993). Rehydration is required for the resumption of the entire metabolic network, including DNA repair that takes place during early imbibition when the embryo cells are at the G1 stage (Bewley 1997; Osborne 2000). In the desert plant *Artemisias sphaerocephala*, imbibed seeds release pectinaceous mucilage that preserves the seed moisture during germination. Interestingly these authors hypothesize that such conditions might promote DNA repair under environmental stress (Yang et al. 2001).

#### DNA repair pathways triggered by seed priming

Controlled rehydration carried out during priming triggers specific DNA repair mechanisms, such as the base excision repair (BER) pathway in which DNA glycosylases remove oxidized bases, generating abasic sites that are subsequently filled through DNA synthesis. The most frequent oxidation product is 8-oxoguanine, removed by either the 8-oxoguanine DNA glycosylase/lyase (OGG1) or formamidopyrimidine-DNA glycosylase (FPG) (Córdoba-Cañero et al. 2014). Upregulation of *OGG1* and *FPG* genes has been reported in hydroprimed and bioprimed *M. truncatula* seeds (Forti et al. 2020a, b; Pagano et al. 2022a), and osmoprimed eggplant (*Solanum melongena* L.) seeds (Kiran et al. 2020). Seed priming triggers DSBs repair through non-homologous end joining (NHEJ) and homologous recombination (HR), as well as mismatch repair (MMR) pathways (Kiran et al. 2020). Upregulation of *SOG1* gene, encoding the master regulator of DDR in plants, was reported along dry-back in hydroprimed *M. truncatula* seeds, indicating active DNA damage-dependent signaling (Pagano et al. 2022a). Despite these pieces of evidence, the current knowledge about DNA repair and genome maintenance mechanisms activated by seed priming still needs to be deeply explored.

#### DNA repair genes as early indicators of the seed response to priming

Highly conserved, specific repair pathways target the different types of DNA lesions such as base- and nucleotide-excision repair (BER, NER) acting on base damage/single-strand breaks (SSBs) and bulky lesions, as well as non-homologous end joining (NHEJ), alternative NHEJ pathways (alt-NHEJ), and homologous repair (HR) involved in the repair of double-strand breaks (DSBs). Such mechanisms have been investigated in the context of pre-germinative metabolism to assess their impact on seed quality, their involvement in the response to priming, and their potential as sources of seed quality hallmarks (Fig. 3). The highly conserved DNA damage checkpoint kinases ATAXIA TELANGIECTASIA MUTATED (ATM) and ATM AND RAD3-RELATED (ATR) are now regarded as crucial determinants of seed viability and their function can be modulated in response to seed dormancy and environmental signals (Waterworth et al. 2016, 2019). DSBs are highly cytotoxic and can cause major karyotypic instability leading to cell death (Amiard et al. 2013).The seed repair response for removing DSBs is triggered during early imbibition, even in high-quality seeds, with a significant role played by DNA ligases (Waterworth et al. 2010). Along with seed imbibition, increased levels of 7,8-dihydro-8-oxoguanine (8-oxodG) can be detected (Macovei et al. 2010, 2011a, 2011b; Balestrazzi et al. 2011). Upregulation of several BER genes, e.g., *OGG1* (8-oxoguanine DNA glycosylase), *FPG1* (formamidopyrimidine-DNA glycosylase), and *TDP1* (tyrosyl-DNA phosphodiesterase) associated with metabolic resumption has been reported in imbibed seeds of *Medicago truncatula* (Pagano et al. 2017, 2019, 2022a; Forti et al. 2020b), *Arabidopsis* (Chen et al. 2012a; Cordoba-Canero et al. 2014), and eggplant (Forti et al. 2020a; Kiran et al. 2020). To date, reports that investigate the use of the expression profiles of DNA repair genes, e.g., BER genes, as early indicators of the seed response to priming have highlighted both genotype- and seed lot-dependent variability (Forti et al. 2020a, 2021).

#### Seed proteome integrity and protein repair/removal: another perspective toward molecular hallmarks

Water uptake triggers the resumption of the functional seed proteome; however, damaged proteins resulting from the desiccation–rehydration cycle need to be repaired or removed. It has been reported that proteins involved in the translation process are preferred targets of the seed protective and repair mechanisms. Issues regarding proteome integrity could disclose new seed quality hallmarks (Fig. 3). The ‘Job’s rule’ states that any other protein can be replaced in imbibed seeds except for those belonging to the translational machinery (Rajjou et al. 2008). Furthermore, proteins associated with the translational apparatus, e.g., ribosomal proteins, are significantly long lived due to peculiar protective mechanisms. They associate with the multi-task LEA (Late Embryogenesis Abundant) proteins acting as shields to prevent damage to the cellular components (Battaglia et al. 2008; Dirk and Downie 2018). Accumulation of LEA proteins has been reported in primed seeds of different species, such as sugarbeet (Capron et al. 2000) and rapeseed (Kubala et al. 2015a). Recurrent damage, resulting from oxidative stress and detected in desiccated as well as aged tissues, is represented by isoaspartate formation. This is the preferred substrate of isoaspartyl methyltyransferase, a repair enzyme active during seed imbibition, particularly on those proteins involved in the processing of the ribosomal RNAs in the nucleolus (Galland and Rajjou 2015; Dirk and Downie 2018). Osmopriming applied to aged tomato seeds was able to restore the activity of isoaspartyl methyltyransferase, suggesting a role of this enzyme in cellular repair during the treatment (Kester et al. 1997). More recently, such a role has been further dissected, revealing that isoaspartyl methyltyransferase repairs isoaspartyl damage occurring in the antioxidant enzymes SOD and CAT, contributing to protection against oxidative stress (Ghosh et al. 2020). Due to the intrinsic properties of seed priming, and the need to keep the balance between stimulation of germination and the undesired side effects caused by excess oxidative injury, proteins involved in repair mechanisms represent promising indicators. The proteome integrity in rehydrating seeds, and specifically the translational machinery components, is also under the control of methionine sulfoxide reductase. The latter prevents the accumulation of oxidized methionine which contributes to decreased translation fidelity (Nelson et al. 2014; Dirk and Downie 2018). The chaperonin peptidyl-prolyl *cis–trans* isomerase, required for proper folding through proline isomerization, and heat shock proteins that protect against misfolding, are included in the set of relevant repair proteins active in germinating seeds (Dirk and Downie 2018). In vivo protein oxidation generates carbonyl groups, an irreversible process leading to the selective 20S proteosome-mediated degradation of carbonylated proteins (Nyström 2005). ROS-induced oxidation of storage proteins during seed imbibition triggers reserve mobilization by promoting proteolytic cleavage, thus supporting the germination process (Job et al. 2005; Barba-Espín et al. 2011; El-Maarouf-Bouteau et al. 2013). Similarly, oxidative modifications of mRNAs are implicated in the early step of seed germination, since the occurrence of modified bases in selected transcripts impairs translation, possibly facilitating dormancy release (El-Maarouf-Bouteau et al. 2013).

#### The emerging contribution of nucleolus to seed priming issues

Metabolic resumption and recovery of translation at the onset of germination are linked to the nucleolar functional restoration since the plant nucleolus is the site of ribosomal RNA synthesis and ribosome biogenesis (Kalinina et al. 2018). Morphological changes (e.g., vacuolization) associated with nucleolar activation have been reported in early studies on germinating embryos (Deltour and de Barsy 1985); however, the current knowledge concerning the role of nucleolus in the context of the pre-germinative metabolism is still scanty. Evidence has been so far provided about the involvement of plant nucleolus in stress signaling pathways, and in the maintenance of genome integrity (Kalinina et al. 2018). Given such findings, the molecular processes occurring within the nucleolus might significantly contribute to seed vigor and take part in the complex response to seed priming (Pagano et al. 2022a). DDR genes implicated in the seed repair response during rehydration have been correlated with nucleolar functions as reported for TDPs (Donà et al. 2013; Macovei et al. 2018). A deeper investigation of those processes connecting ribogenesis and DDR will provide valuable knowledge for applied research purposes, as discussed below (see overpriming). It is worth noting that the knowledge on nucleolar function has been so far gathered using *Arabidopsis* as a model plant, whereas a few studies dealing with pre-germinative metabolism have been performed in the model legume *M. truncatula.* Although promising, such data need to be validated in a larger panel of model and crop plants. Furthermore, the potential of the nucleolus as a source of novel seed quality hallmarks (Fig. 3) still needs to be deeply explored.

The current scientific literature reflects the efforts made by the plant research community to dissect the plethora of molecular events underpinning the seed response to priming and clarify those mechanisms that promote the seed's ability to repair stress-induced damage. The complexity of the pre-germinative metabolism is delaying the current research addressing one of the main open questions listed in Fig. 1: *What is the contribution of seed repair response in germination and priming?* The seed priming techniques remain too empirical and the frequency of undesired side effects, that compromise the successful outputs of treatments, is far from being acceptable in terms of economic value and competitiveness of the final product. The availability of additional experimental systems will help expand the current knowledge of the drawbacks of seed priming.

### Exploring the drawbacks of seed priming

When does seed priming fail? A frequent drawback is overpriming that compromises survival to desiccation (dry-back step), but a relevant issue is also the reduced shelf-life of the primed seeds (Wang et al. 2018, Zulfiquar 2021, Tu et al. 2022). A systematic analysis of the molecular events occurring in seeds subjected to non-optimal priming treatment is missing. In addition, the strong genotype- and seed lot-dependent variability represents a major concern since it delays the search for common hallmarks of seed quality. This section provides a critical review of the current literature dedicated to overpriming and post-priming storage.

#### Overpriming compromises survival to dry-back

Among the parameters that should be kept under control, there is the duration of the rehydration step. When the treatment is prolonged, the resulting metabolic acceleration can easily bring seeds to overcome the critical threshold that leads to irreversible germination (Fig. 2). When such a condition occurs, desiccation tolerance is lost and embryos will not survive the dry-back step. This process, known as overpriming, can significantly compromise the efficacy and economic value of priming protocols. Interestingly, it is also known that desiccation tolerance can be rescued within a short developmental window between germination and seedling establishment when seeds are challenged with osmotic stress (Bruggink and van der Toorn 1995; Peng et al. 2017). It has been suggested that the resumption of desiccation tolerance can help seeds facing unpredicted drying conditions soon after germination (Dekkers et al. 2015). In *M. truncatula*, this crucial developmental window exists only when the length of the protruding radicle ranges between 1 and 3 mm (Buitink et al. 2003; Maia et al. 2011), and it has been exploited to establish an experimental system to investigate the role of antioxidant response and DNA repair pathways in primed *versus* overprimed *M. truncatula* seeds as well as the molecular events associated with the progressive loss of desiccation tolerance (Pagano et al. 2022a,b). The focus on the role of nucleolus and ribogenesis in such a context of enhanced oxidative stress and genotoxic damage allowed disclosing of novel potential molecular players linked to dehydration-induced genotoxic stress. Nucleolar perturbation resulted in increased accumulation of precursor and mature rRNAs, detected only in overprimed embryos along the dry-back step. Among the tested rRNA, precursor and mature 5.8S rRNA showed an early peak at 2 h of dry‐back only in overprimed embryos, suggesting a potential role as a stress hallmark concerning overpriming (Pagano et al. 2022a). Although a similar profile was also reported by the same authors in overprimed seeds of the closely related legume alfalfa (*Medicago sativa* L.), validation of 5.8S rRNA as a stress hallmark, at least in legumes, will necessitate testing across a wider range of species and/or genotypes.

#### Conditions leading to overpriming change with species, cultivars, and seed lots

The use of different model systems becomes a necessary premise to gain insights into the associated events. Cotyledons, hypocotyls, and radicles display different levels of desiccation tolerance, while the aerial parts are the most desiccation tolerant (Reisdorph and Koster 1999; Dekkers et al. 2015). It is also known that desiccation tolerance can be improved by applying osmotic agents or phytohormones (Reisdorph and Koster 1999; Buitink et al. 2003). Furthermore, it is essential to consider the variability of different subpopulations within a seed lot in their response to priming and dry-back. Such issues are reflected in one of the main open questions listed in Fig. 1: *How to tackle inter- and intra-specific variability in assessing and improving seed quality?* Pagano et al. (2022b) investigated the occurrence of mild/severe overpriming in *M. truncatula* seeds, testing kinetin-mediated hormopriming as a tool to counteract overpriming and using ROS accumulation during dry-back as a potential hallmark of post-priming seedling establishment performances. A significant increase in the percentage of seedlings with aberrant morphology positively correlated with the increased radicle length measured before dry-back. Kinetin-mediated hormopriming was not able to rescue overpriming; however, it could accelerate and synchronize germination and this effect could represent an alternative route to decrease the exposure to desiccation stress within a tailored priming protocol (Pagano et al. 2022b). On the other hand, prolonged exposure to kinetin results in reduced radicle growth, associated with global metabolomic depletion and accumulation of DSBs (Araujo et al. 2019). A direct correlation was evidenced between radicle length measured before dry-back, ROS levels quantified at the end of dry-back, and the occurrence of ‘aberrant’ seedling morphology. Both spectrophotometric assays and DAB staining highlighted ROS accumulation in *M. truncatula* embryos during post-priming dry-back, and similar profiles were also observed in *M. sativa* (Pagano et al. 2022b). Such findings suggest the possibility of using ROS as hallmarks of seed priming progression, at least in these two closely related legumes. Such a case study could be reproduced in horticultural model/crop species and/or related CWRs, according to the need to explore different models of pre-germinative metabolism.

#### Storage potential is decreased in primed seeds

Another major drawback of seed priming is the limited shelf-life of the treated seeds (Fig. 2), observed in most cases (Nath et al. 1991; Owen and Pill 1994; Bray 1995; Hussain et al. 2015; Yan 2017), although beneficial effects on seed storability are also documented (Burgass and Powell 1984; Georghiou et al. 1987; Dearman et al. 1986; Abnavi and Ghobadi 2012; Pandey and Pati 2017; Das and Dutta 2022). Specific case studies are reported for cotton seeds subjected to mannitol-mediated osmopriming able to maintain enhanced germination performance for at least 12 months of storage at room temperature, thus having an added value for commercial purposes (Toselli and Casenave 2014). Interestingly, priming applied to low-vigor seeds boosts the repair of damaged structures and improves the storage potential whereas the physiological acceleration triggered in high-vigor seeds can be deleterious to longevity (Powell et al. 2000). Both dry-back and storage conditions affect post-priming seed longevity; a rapid dehydration step may alter the levels of soluble carbohydrates, impairing desiccation tolerance whereas slow dehydration may improve the storability of primed seed (Bruggink et al. 1999). Several environmental factors, namely oxygen availability, moisture, and temperature, are regarded as key players in seed deterioration. Increased oxygen levels promote cellular respiration and, combined with high temperature and relative humidity, accelerate seed deterioration (Ellis et al. 2008; Schwember and Bradford 2011). Such conditions trigger ROS accumulation leading to oxidative damage, loss of membrane integrity, depletion of seed reserves, and loss of seed viability (Liu et al. 2016). The impact of these factors on the longevity of primed seeds is still poorly investigated and this reminds that there is still an open question in applied research: *How to overcome the reduced storability of primed seeds?* (Fig. 1).

#### How to maintain the priming benefits in stored seeds?

Improved knowledge of the cellular and molecular events associated with the different storage conditions applied to primed seeds is required to support the technology. Primed seeds are more prone to oxidative damage compared to non-primed seeds (Hussain et al. 2015). Reduced starch metabolism in primed rice seeds has been correlated with decreased germination; however, it has been suggested that, despite reserve depletion, seeds still retain metabolic energy for germination (Justice and Bass 1978). Vacuum storage is beneficial to primed seeds possibly because they are kept under low moisture content and limited respiration (Chiu et al. 2003; Yeh et al. 2005; Hu et al. 2006; Feng et al. 2017; Wang et al. 2018). The metabolic acceleration imposed by priming and the related repair activities triggers cell cycle progression. Indeed, the screening of biologically active compounds able to suppress *Arabidopsis* seed deterioration revealed that treatments with the cell cycle inhibitor mimosine and other molecules with similar roles (aphidicolin, hydroxyurea, and oryzalin) were able to improve seed storability (Sano and Seo 2019). Priming with antioxidants has been also tested as a strategy to prolong rice and oat seed viability during storage (Xu et al. 2020; Xia et al. 2020). The poor performance of primed, desiccation-tolerant orthodox seeds under storage resembles the response of desiccation-sensitive, recalcitrant seeds. It has been reported that recalcitrant seeds do not produce/activate components/mechanisms that typically provide desiccation tolerance in orthodox seeds (Berjak and Pammenter 2013). For instance, the absence of LEA proteins in recalcitrant seeds of *Castanospermun australe* turned out to be a crucial determinant of desiccation tolerance (Delahaie et al. 2013). Looking at the molecular dynamics occurring in recalcitrant seeds might bring knowledge useful to unravel the response to storage observed in primed orthodox seeds. In this context, basic research should be expanded in both model and crop species.

Farmers need to know how long the primed seeds can retain high germinability before planting to properly manage their seed lots and companies are committed to providing such information. However, the seed potential to survive long-term storage differs among cultivars and species; thus, one possible strategy to overcome this drawback would be the selective use of genotypes with enhanced storage potential. Improving such a trait will increase the commercial value of primed seeds as the farmers will be able to manage sustainably their field activities when challenged by deleterious climate conditions or unexpected drawbacks. Moreover, decoding the genetic and physiological traits underlying the storability of primed seeds may lead to the development of targeted assays to monitor and prevent seed deterioration in different crop species, with beneficial effects on seed marketing and distribution.

#### Protocols for effective storage of primed seeds

It is difficult to list guidelines for improving the viability of stored primed seeds. Several parameters should be considered carefully, e.g., storage time should be defined based on the seed ageing profile, priming and conservation conditions should be adjusted to buffer genetic and environmental variability. Tu et al. (2022) reported that the priming effects were maintained in pepper seeds preserved in sealed plastic bags at a low temperature. Priming with ascorbic acid and sodium nitroprusside allowed an improved performance of stored sunflower seeds, possibly due to a boost in the antioxidant defense (Pereira et al. 2022). Priming with water and spermidine, followed by drying in presence of silica gel and a saturated saline solution was applied to tobacco seeds that were cryopreserved in liquid nitrogen for 24 h without loss of germinability (Lopes et al. 2018). These findings suggest that the longevity of primed seeds can be improved by selecting optimal priming parameters, storage, and post-storage conditions (Fabrissin et al. 2021).

#### Priming memory in seeds

The concept of priming memory has been used to explain the beneficial effects of seed priming in terms of enhanced stress tolerance at the seed/seedling level (Srivastava and Kumar 2021). Seeds are brought toward an advanced physiological stage while experiencing abiotic stresses that trigger stress-responsive mechanisms. This provides the basis for cross-tolerance, a feature maintained notwithstanding dry-back (Chen and Arora 2013). There is increasing evidence that seed priming stimulates the plant's immune memory, a feature held through development or even across generations (Yang et al. 2022). The molecular events associated with stress memory *in planta* have been explored at the level of chromatin remodeling, alternative transcript splicing, metabolite accumulation, and autophagy; thus, similar processes might also contribute to establishing stress memory during seed priming (Liu et al. 2022). Given that stress memory is a valuable feature ascribed to seed priming, another research question arises: *To what extent can stress memory contribute to maintaining enhanced stress tolerance in primed seeds subjected to storage?* (Fig. 1). Priming memory is influenced by several factors, including priming agents, dry-back, and storage conditions, as well as seed lot quality (Sano et al. 2017; Fabrissin et al. 2021). Primed seeds can survive storage but lose the high germination capacity acquired with treatments. Different profiles describing the persistence of such benefits during storage have been so far reported, revealing variable responses that make it difficult to figure out guidelines useful for seed operators. Osmoprimed leek seeds maintained the added value of the treatment during storage in silica gel for up to 15 months (Corbineau et al. 1994) whereas osmoprimed *B. napus* seeds showed superior germination performance after 6 months of storage at 8 °C (Basra et al. 2003). *Capsicum frutescens* seeds primed with liquid seaweed sap of *Kappaphycus alvarezii* and *Gracilaria edulis* maintained their high vigour index after 12 months of storage at room temperature (Dutta et al. 2019), similar to what was reported in pepper (Sivritepe and Sivritepe 2008) and tomato (Hérnadez-Herrera et al. 2014). It has been suggested that the enhanced mineral content and phytohormone levels of seaweed extracts might contribute to preserving the priming benefits along storage (Layek et al. 2015). These benefits were lost rapidly in the same seeds subjected to hydropriming (Corbineau et al. 1994; Basra et al. 2003; Dutta et al. 2019). The molecular and physiological bases that support the ability of primed seeds to keep their enhanced vigour under storage are still poorly investigated, although there is evidence that different protective mechanisms are triggered in response to different priming treatments (Fabrissin et al. 2021).

Can failure help us understand the keys to successful treatments? For sure there is still much to learn from seeds challenged with irreversible damage. In this context, open questions can be better assessed using and/or integrating up-to-date tools that often need to be adapted to the unique features of the seed tissues.

### Tools to investigate the impact of seed priming on the pre-germinative metabolism

This paragraph provides an update on the techniques currently used to monitor seed vigor at different levels, by capturing key parameters from the morphological to the molecular point of view. Considering the complexity of the seed vigor trait, all these approaches offer unique advantages and, in most cases, they should be integrated into multidisciplinary strategies to address some of the main open research questions listed in Fig. 1: *How to identify reliable hallmarks of seed quality? How to tackle inter- and intra-specific variability in assessing and improving seed quality? How to enhance the potential of underutilized germplasm resources?*

#### Digital phenotyping

The first level for assessing the impact of seed priming is the accurate phenotyping of the germination process. Scoring can be carried out by operators through visual observation; however, this affects accuracy and for this reason, several tools, e.g., Germinator (Joosen et al. 2010), *pheno*Seeder (Jahnke et al. 2016), MultiSense (Keil et al. 2017), SeedGerm (Colmer et al. 2020), ScreenSeed (Merieux et al. 2021), have been so far developed to automatize seed diagnostics and associated phenotypic analysis. Spatiotemporal dynamics of seed germination can be measured through digital analysis based on seed color, texture, morphology, and growth patterns, with the added value of standardization. The Germinator software package allows high-throughput scoring of germinating seeds in transparent trays stacked in an incubator (Joosen et al. 2010), whereas the *pheno*Seeder system relies on a pick-and-place robot and a modular setup of sensors that provide biometric traits from individual seeds and calculate three-dimensional data (Jahnke et al. 2016). The technological platform known as MultiSense tool allows the parallel monitoring of respiration in imbibing seeds (up to 100 samples) over an extended period, tracking oxygen (O_2_), carbon dioxide (CO_2_), and/or pH (Keil et al. 2017). The ScreenSeed technology, developed for *Arabidopsis*, provides a fast procedure allowing to handle thousands of seeds without compromising the repeatability or accuracy of the germination measurements (Merieux et al. 2021). An approach for determining seed quality was developed using FT-NIR spectroscopy and X-ray imaging data by FT-NIR spectroscopy that can be used in conjunction with machine learning algorithms to improve seed germination and vigor prediction (Dantas de Medeiros et al. 2020).

#### Genotoxicity assessment

As previously highlighted, the issues of genotoxic stress arising along the seed life cycle and peaking during rehydration are crucial in terms of seed vigor. The ability to remove DNA damage is regarded as an excellent component of such a complex trait; thus, the availability of reproducible, standardized tests for genotoxicity assessment in germinating seeds is highly desirable. Such tools are regarded as extremely valuable when exploring the seed response to stress conditions possibly imposed by novel priming treatments. Comet assay (single-cell gel electrophoresis, SCGE) is widely used to measure not only DNA damage accumulation (SSDs, DSBs, oxidized base lesions) but also the dynamics of DNA repair (Olive and Banath 2006, Ventura et al. 2013; Collins 2015). This is a sensitive and low-cost method, widely utilized for both basic and applied research purposes, and diagnostics. The comet assay can be performed under alkaline conditions to detect total DNA strand breaks, including those generated at the apurinic or apyrimidinic sites (AP sites, alkali-labile sites, ALS) whereas the neutral version specifically detects the presence of DSBs (Collins et al. 2008). The technique has been optimized for its application on seed tissues, e.g., at the radicle protrusion stage, as reported by Pagano et al. (2017, 2019) who evaluated the genotoxic effects of the histone deacetylase inhibitors trichostatin A and sodium butyrate in *M. truncatula* by performing alkaline comet assay. The same approach was also used by de Sousa Araujo et al. (2019) to disclose the genotoxic impact of prolonged kinetin-based seed priming in *M. truncatula*. Genomic instability was evaluated in seeds of rice and beans stored in gene banks using comet assay to investigate the molecular bases of seed deterioration (Dantas et al. 2018). Comet assay has been tested on embryos or embryo axes isolated from eggplant (Kiran et al. 2020), *M. truncatula* (Pagano et al. 2022a), and *Acer pseudoplatanus* L. (Plitta-Michalak et al. 2022) seeds.

#### Detection of reactive oxygen species

The pivotal role of ROS as players able to influence the delicate balance between stimulation of the germination process (signal molecules) and oxidative damage (cytotoxic effect) makes their detection and quantification a relevant step in the assessment of seed quality, especially in the context of seed priming. ROS detection is challenging due to its low concentration and short half-life (Steffens et al. 2013). Indirect measurement can be carried out by quantifying lipid peroxidation expressed as malondialdehyde (MDA) content in dry and imbibed seeds, as reported in studies describing the impact of seed priming in sunflower (Bailly et al. 2000), cucumber (Krainart et al. 2015), barley (Junhaeng et al. 2018), wheat (Hameed et al. 2019), rice (Akhila and Puthur 2020), *Solanum villosum* (Forti et al. 2021). Histochemical approaches are also used to detect ROS, e.g., 3,3-diaminobenzidine (DAB) (Thordal-Christensen et al. 1997) that reacts with H_2_O_2_ in a peroxidase-catalyzed reaction producing an oxidized insoluble brown precipitate, and nitroblue tetrazolium (NBT) useful to detect the superoxide radical (Fryer et al. 2002). DAB and NBT assays have been used to characterize the response of seeds treated with osmopriming (Kiran et al. 2020), hydropriming and hormopriming (Pagano et al. 2022b; Lee et al. 2022), nanopriming (Mahakham et al. 2017; Li et al. 2022b). Fluorescent probes, such as derivates of dichlorodihydrofluorescein diacetate (DCFH-DA) can be used as a non-invasive approach to detect ROS through live-cell imaging (Kristiansen et al. 2009; Bailly and Merendino 2021) or specific fluorimetric assays (Doria et al. 2019; Macovei et al. 2019; Forti et al. 2020a, 2021; Pagano et al. 2022a, b). ROS are detected by EPR using spin traps or spin probes with different properties including lipophilicity, reaction kinetic, and stability of adducts (Buitink et al. 1999, Bacic et al. 2008, Araujo et al. 2016, 2019; Pagano et al. 2017, 2022a). EPR has proved an informative approach when used to explore the seed response to priming (Roqueiro et al. 2012; Pagano et al. 2022a).

#### Omics

Omics-based analyses can speed up the search for reliable indicators since they provide global, enlarged datasets, where bioinformaticians dig deeply working side by side with seed biologists (Macovei et al. 2017). Proteomic analyses, used to assess the impact of priming on seed metabolism from a global perspective, have highlighted the functional networks boosted through controlled rehydration, to support accelerated germination. Enzymes of the glycolysis pathway and TCA cycle are activated to provide the required energy as well as antioxidant enzymes and enzymes involved in the synthesis of antioxidant compounds, along with enzymes required for fatty acid, amino acid, and protein synthesis (Yacoubi et al. 2011; Lv et al. 2016). Proteins participating in water transport, cell wall modification, cytoskeletal organization, and cell division were also differentially accumulated in primed seeds (Kubala et al. 2015b). Findings resulting from metabolomics-based investigation reflect the high variability in sugar metabolic pools and interconversion pathways existing in phylogenetic distant plant families and underline a relevant issue. The dynamic changes of the seed pre-germinative metabolism should be taken into account, more that the profile of every single metabolite (He et al. 2019; Domergue et al. 2019). Omics are also used to investigate the impact of cumulative rehydration–dehydration cycles. Analysis of protein accumulation in *Ferocactus peninsulae* seeds challenged with rehydration–dehydration cycles revealed differential expression of proteins involved in primary metabolism, ubiquitination pathways, and reserve protein. Changes in total RNA stability were also observed (López-Urrutia et al. 2014). A link between seed respiration, energy metabolism, fatty acid β-oxidation, nitrogen mobilization, membrane permeability, and enhanced germination performance was highlighted in seeds of the *Arabidopsis* ecotype *Ler* subjected to recurrent rehydration–dehydration cycles (Bai et al. 2012). Noteworthy, although single-case studies bring valuable information, only systematic and expanded investigations covering a wide range of species will contribute to significant advances with broad implications.

### Hallmarks of successful seed priming

#### The concept of seed quality hallmark

At the basis of priming treatments, the rehydration–dehydration cycle can be described in terms of metabolic pathways that are triggered, modulated, or turned off, depending on the seed's physiological stage. Understanding the way seed priming affects, either positively or negatively, such metabolic pathways and impacts gene expression and protein/metabolite accumulation/depletion represents an essential step toward the identification of novel seed quality hallmarks. The awareness that enhanced seed vigor stands as the starting point of a robust climate-resilient agrifood chain has prompted researchers to look at the basic aspects of the seed pre-germinative metabolism through fresh eyes, building an updated concept of seed quality biomarkers or hallmarks (Corbineau 2012; Jeevan Kumar et al. 2016; Domuerge et al. 2019). The development of effective hallmarks of the seed response to priming, nowadays one of the main issues for seed companies, can be achieved through a deeper understanding of the molecular events associated with the seed pre-germinative metabolism. Such events need to be dissected at different levels with integrated experimental approaches, to provide a full picture from which crucial players could be retrieved and classified as potential quality hallmarks. Once again, the main open questions to be considered are: *What is the contribution of seed repair response in germination and priming? How to identify reliable hallmarks of seed quality? How to tackle inter- and intra-specific variability in assessing and improving seed quality?* (Fig. 1). It is generally acknowledged that such potential seed quality biomarkers can be found only by looking at the plethora of metabolic pathways (e.g., antioxidant response, DNA repair, RNA- and protein-related processes), activated by imbibition, that contribute to defining seed quality (Rajjou et al. 2012). Seed quality hallmarks can be represented by the presence/absence or specific accumulation/depletion patterns of proteins and metabolites, as well as by peculiar gene expression profiles. They can be assigned to the different steps along the rehydration–dehydration cycle (Fig. 2).

#### Do we have reliable seed quality hallmarks?

Once a potential hallmark has been identified, validation should follow by testing the occurrence of that specific biomarker in a range of low- and high-quality seed lots of different genotypes of the same species or even in different species. However, the strong genotype-dependent variability frequently observed during validation assays significantly delays the process. A list of potential hallmarks of seed vigor is provided in Table 1 and discussed. Enhancement of DNA repair pathways during priming can be traced by monitoring the expression levels of DDR genes, such as *RAD3* and *RAD54* (Sharma and Maheshwari 2015), *OGG1,* and *FPG* genes (Forti et al. 2020a, 2020b; Pagano et al. 2022a, 2022b). Accumulation of β tubulin has been used as an indicator of priming-mediated metabolic acceleration (Lanteri et al. 2000) whereas the group 2 LEA protein dehydrin has been proposed as a potential biochemical marker of priming-mediated stress tolerance (Chen et al. 2012b). Comparative proteomics carried out on hydroprimed sugarbeet seeds has revealed distinctive proteins upregulated during priming and downregulated during aging, revealing the glyoxylate enzyme isocitrate lyase as a putative contributor to seed vigor (Catusse et al. 2011). Galactinol levels increase during seed maturation and subsequently decline during germination in chickpeas (Salvi et al. 2016), whereas galactinol accumulation results in detrimental effects on seed quality in maize (Li et al. 2017b). The role of galactinol as a marker for seed longevity was also proven (De Souza Vidìgidal et al. 2016). Metabolic biomarkers of seed quality include galactose and gluconic acid, negatively correlated with seed vigor in rice (Chen et al. 2022). Changes in polyamine accumulation, reported in *M. truncatula* seeds facing genotoxic stress, highlight their potential as metabolic signatures of seed germination under adverse conditions (Pagano et al. 2019). Hu et al. (2018) analyzed the effects of rehydration–dehydration cycles by looking at membrane glycerolipids; total glycerolipids remained constant across the cycles but phosphatidic acid and diacylglycerol showed opposite accumulation profiles. As the number of cycles increased, deleterious metabolic changes (increased lipid unsaturation, decreased levels of plastidic lipids, and phosphatidylserine acyl chains lengthened) were concomitant with loss of seed viability (Hu et al. 2018).

**Table 1:** Putative molecular hallmarks of the seed response to priming

Type	Name	Function	Plant species	Seed priming	Stress treatment	Methodology	References
G	*CAP85*	Response to desiccation stress	*S. oleracea* L	Osmopriming	–	QRT-PCR	Chen et al. (2012a, b)
	*P5CS*	Proline biosynthesis	*B. napus* L	Osmopriming	–		Kubala et al. (2015a, b)
	*RAD3*, *RAD54*	DNA repair (NER, HR)	*C. arietinum* L	Hydropriming Osmopriming	–		Sharma & Maheshwari (2015)
	*OGG1*, *LIGIV*	DNA repair (BER)	*M. truncatula* L	–	TSA-induced genotoxic stress		Pagano et al. (2017)
	*ALKBH1, APE1, FEN1, LIG1* *SOD, MT2, TRH1* *SPDS, SPMS*	DNA repair (BER) ROS scavenging Polyamine biosynthesis	*M. truncatula* L	–	NaB-induced genotoxic stress		Pagano et al. (2019)
	*TPS1*, *TPS7*, *TPS10*, *TPPA*, *TPPI*, *TRE*	Threalose metabolism	*M. truncatula* L	–	Osmotic/salt stress		Macovei et al. (2019)
	*OGG1*, *FPG1* *SOD, APX*	DNA repair (BER) ROS scavenging	*S. melongena* L	Hydropriming Osmopriming	–		Forti et. al 2020a Kiran et al. (2020)
	*FPG1* *SOD, APX, MT2*	DNA repair (BER) ROS scavenging	*M. truncatula* L	Hydropriming Biopriming	–		Forti et al. (2020b)
	5.8S rRNA	Ribogenesis	*M. truncatula* L *M. sativa* L	Hydropriming	Desiccation stress Overpriming		Pagano et al. (2022a, b)
P	β-Tubulin	Microtubules, DNA replication	*C. annuum* L	Osmopriming	–	Immunoblotting	Lanteri et al. (2000)
	Dehydrin CAP85	Response to desiccation stress	*S. oleracea* L	Osmopriming	–		Chen et al. (2012a, b)
	Isocitrate lyase	Reserve mobilization	*B. vulgaris* L	Hydropriming	–	Proteomics	Catusse et al. (2011)
	Galactinol synthase	RFOs synthesis	*A. thaliana*	–	Accelerated aging	Enzyme assay	Li et al. (2017a, b)
M	Galactinol	Osmoprotectant	*C. arietinum* L	–	Storage	GC-FID, GFC	Salvi et a. (2016)
	Galactinol	Osmoprotectant	*Z. mays* L	–	Accelerated aging		Li et al. (2017a, b)
	Phosphatidic acid, diacylglycerol	Membrane integrity	*A. thaliana* *L. perenne* L *N. tabacum* L	–	Desiccation stress	Lipidomics	Hu et al. (2018)
	Polyamines	ROS scavenging	*M. truncatula* L	–	NaB-induced genotoxic stress	Metabolomics	Pagano et al. (2019)
	Galactose, gluconic acid	RFOs metabolism	*O. sativa* L	–	Accelerated aging		Chen et al. (2022)

For each hallmark, the corresponding function and the plant species investigated are shown. The seed priming technique and/or stress treatment imposed to seeds that allowed to disclose the hallmark, as well as the methodology used are shown

*ALKBH* alkylation repair homolog, *APE* apurinic/apyrimidinic endonuclease, *APX* ascorbate peroxidase, *FEN* flap endonuclease, *FPG DNA* formamidopyrimidine DNA glycosylase, *G* gene, *GC*-*FID* gas chromatography-flame ionization detector, *GFC* gel filtration chromatography, *LEA* late embryogenesis abundant, *LIG* ligase, *M* metabolite, *MT* metallothionein, *NaB* sodium butyrate, *OGG* oxoguanine *DNA* glycosylase, *P* protein, *P5CS* pyrroline-5-carboxylate synthase, *QRT*-*PCR* quantitative real-time polymerase chain reaction, *RAD* radiation-sensitive, *RFO* raffinose family oligosaccharides, *SOD* superoxide dismutase, *SPDS* spermidine synthase, *SPMS* spermine/spermidine synthase, *TRH* thioredoxin, *TSA* trichostatin, *TPS* trehalose phosphate synthase, *TPP* threalose phosphate phosphatase, *TRE* trehalase

### Conclusion

The renewed potential of seed priming to overcome severe drawbacks is particularly visible when seeds are used in fragile agroecosystems (Singh et al. 2020; Devika et al. 2021). This leads to further open questions: *How to enhance the potential of underutilized germplasm resources?* (Fig. 1). Seed priming promotes the repair of drought-induced damage, supporting the recovery of drought-stressed plants when water becomes available (Marthandan et al. 2020; Aswathi et al. 2021). The use of “on-farm” seed priming is effective against soil crusting and limited soil moisture, typical of the semi-arid areas of developing countries, and improves disease tolerance (Harris et al. 2005; Carrillo-Reche et al. 2018). Cost-effective and easy-to-manage “on-farm” seed priming represents a unique route toward food security in the poorest regions of the planet.

Despite this promising scenario, certain relevant research questions need to be assessed to ensure the transition of seed priming toward a new technological phase. In this review, such open questions have been underlined in association with the different topics and/or problematics that constitute state-of-the-art research on seed priming. The complexity of seed physiology, the genetic background, and the influence of the environment are the main sources of variability when the technique is applied. Researchers must investigate such variability to find the key players contributing to the delicate balance between the stimulation of seed metabolism and the undesired side effects (e.g., oxidative damage and genotoxic stress) caused by suboptimal treatments. Would it be possible to identify universal hallmarks of the seed response to priming useful or would be more reasonable to think about indicators restricted to specific taxonomic groups, e.g., legumes? To what extent storability of primed seeds can be improved? These are the challenges of seed priming, already taken up by the scientific community. Such challenges should be taken up and addressed, also considering the feasibility of seed priming at the field level. When dealing with fragile ecosystems, the choice of the most appropriate priming approach should be carefully handled to maximize the benefits. Participatory trials with local farmers should help seed technologists and researchers in the validation of candidate treatments.

Given the current advances in seed priming and the efforts made to understand the molecular dynamics underlying either the benefits or the drawbacks of this technique, which recommendations should be given to the seed operators (e.g., technologists, breeders) of the private and public sectors? Is it possible to draw some guidelines? Although promising results need to be consolidated through the extensive validation of novel hallmarks, seed technologists should be more focused on finding out novel treatments, working on combinations of priming agents, and exploiting new types of materials.

## Data Availability

Not applicable.
